# Dissemination of a COVID-19 Rapid Response Telehealth Group Addressing Worry and Social Isolation Among Older Adults

**DOI:** 10.1093/geroni/igab046.2181

**Published:** 2021-12-17

**Authors:** Rachel Weiskittle

**Affiliations:** University of Colorado Colorado Springs, Colorado Springs, Colorado, United States

## Abstract

In response to the urgent need for virtual mental health treatments during the COVID-19 pandemic, an 8-week group intervention deliverable over video or telephone was developed and disseminated in March 2020. Manual content addressed social isolation and information related to COVID-19. In August 2020, a national web-based provider feedback survey was disseminated to evaluate feasibility of the manual. Respondents (n = 21) across a variety of geriatric mental health clinics reported this intervention to be effective and clinically useful with their patients in providing social support and in mitigating COVID-19 anxieties. The majority of respondents delivered the group in multiple cohorts and found the manual adaptable beyond the early pandemic period.

